# Supplementary material for: The Systems Biology Research Tool: evolvable open-source software
Source: BMC Syst Biol. 2008 Jun 29;2:55. doi: 10.1186/1752-0509-2-55 (PMC2446383; doi:10.1186/1752-0509-2-55)
Supplement: Additional file 1 — SBRT Archive. An archive of the current version of the Systems Biology Research Tool. [file 1752-0509-2-55-S1.zip › sbrt-1.4.0/doc/developers_guide/api/sbrt/shell/text/math/CorrResFormatterV1.html]

CorrResFormatterV1


|  |  |  |
| --- | --- | --- |
| **Overview** \| **Package** \| **Class** \| **Use** \| **Tree** \| **Deprecated** \| **Index** \| **Help** | |  |
| **PREV CLASS**   **NEXT CLASS** | **FRAMES**    **NO FRAMES**     **All Classes** |  |
| SUMMARY: NESTED \| FIELD \| CONSTR \| METHOD | DETAIL: FIELD \| CONSTR \| METHOD |  |


---


## sbrt.shell.text.math Class CorrResFormatterV1

```
java.lang.Object
  sbrt.shell.text.math.CorrResFormatterV1
```

**All Implemented Interfaces:**: Formatter<TauResult>, CorrResultsFormatter<TauResult>

---

``` public final class CorrResFormatterV1 extends java.lang.Object implements CorrResultsFormatter<TauResult> ```

This class is a concrete implementation of
`CorrResultsFormatter`. The string representation of
a correlation results is: `Statistic = a | Z = b | n = c`, where
`a` is the computed statistic, `b` is the approximated Z value,
and `c` is the sample size.

**Author:**
:   This class was written and documented by
    Jeremiah Wright while in the Wagner lab.

---

**Constructor Summary**

`CorrResFormatterV1()`
  
          Constructs a new correlation results formatter.


| **Method Summary** | |
| --- | --- |
| `java.lang.String` | `format(TauResult results)`             Returns a formatted string representation of the provided correlation results. |

**Methods inherited from class java.lang.Object**

`clone, equals, finalize, getClass, hashCode, notify, notifyAll, toString, wait, wait, wait`

**Constructor Detail**

### CorrResFormatterV1

```
public CorrResFormatterV1()
```

:   Constructs a new correlation results formatter.


**Method Detail**

### format

```
public java.lang.String format(TauResult results)
```

:   Returns a formatted string representation of the
    provided correlation results.

    :   **Specified by:**: `format` in interface `Formatter<TauResult>`
    :   **Parameters:**: `results` - the results to be formatted. **Returns:**: a formatted string representation of the provided coefficient. **Throws:**: `java.lang.NullPointerException` - if the provided coefficient is `null`.


---


|  |  |  |
| --- | --- | --- |
| **Overview** \| **Package** \| **Class** \| **Use** \| **Tree** \| **Deprecated** \| **Index** \| **Help** | |  |
| **PREV CLASS**   **NEXT CLASS** | **FRAMES**    **NO FRAMES**     **All Classes** |  |
| SUMMARY: NESTED \| FIELD \| CONSTR \| METHOD | DETAIL: FIELD \| CONSTR \| METHOD |  |


---
